# Irritable Bowel Disease: Think Malonic and Methylmalonic Aciduria (CMAMMA)!

**DOI:** 10.1002/jmd2.70119

**Published:** 2026-08-23

**Authors:** Khaled Alatibi, Martin J. Hug, Sara Tucci

**Affiliations:** ^1^ Pharmacy Medical Center—University of Freiburg Freiburg Germany; ^2^ Department of Prosthetic Dentistry, G.E.R.N. Research Center for Tissue Replacement, Regeneration & Neogenesis Medical Center—University of Freiburg Freiburg Germany

**Keywords:** ACSF3, case report, CMAMMA, fatigue, irritable bowel syndrome, juvenile onset, lipoylation degree, pyruvate oxidation

## Abstract

Combined malonic and methylmalonic aciduria (CMAMMA; OMIM 614265) is a rare disorder of mitochondrial fatty acid synthesis caused by pathogenic variants in the ACSF3 gene, which encodes malonyl‐CoA synthetase. The clinical presentation is highly heterogeneous and may include seizures, memory impairment, psychiatric manifestations, and cognitive decline. We describe an adult patient with long‐standing unexplained fatigue and irritable bowel syndrome who remained undiagnosed for 15 years. Subsequent blood and urine analyses revealed elevated methylmalonic acid levels, and molecular genetic testing identified the biallelic ACSF3 variant c.1672C>T (p.Arg558Trp), confirming the diagnosis of CMAMMA. Functional studies in patient‐derived fibroblasts demonstrated altered global protein malonylation together with markedly reduced lipoylation of pyruvate dehydrogenase complex and α‐ketoglutarate dehydrogenase, consistent with impaired mitochondrial energy metabolism. Introduction of a diet enriched in carbohydrates and restricted in protein, based on recommendations for methylmalonic aciduria, resulted in a pronounced worsening of gastrointestinal symptoms, which improved after discontinuation of the dietary intervention. This case highlights that CMAMMA may remain unrecognized for many years and should also be considered in adults presenting with unexplained gastrointestinal symptoms. Furthermore, the findings suggest that impaired glucose oxidation plays a central role in disease pathophysiology and that high dietary carbohydrate intake may exacerbate clinical manifestations.

## Take Home Message

CMAMMA is a clinically underrecognized disorder in which ACSF3 deficiency drives progressive mitochondrial dysfunction through impaired lipoylation, and whose dietary management may require disease‐specific recommendation.

## 1 Introduction

Combined malonic and methylmalonic aciduria (CMAMMA) is an inherited metabolic disorder caused by pathogenic variants in the *ACSF3* gene [1]. *ACSF3* encodes the mitochondrial enzyme malonyl‐CoA synthetase, which converts malonic acid to malonyl‐CoA, the starter substrate of mitochondrial fatty acid biosynthesis (mtFAS) [1]. CMAMMA is biochemically characterized by accumulation of malonic acid (MA) and methylmalonic acid (MMA) in body fluids, with normal malonyl‐CoA decarboxylase activity and mild lactic and pyruvic aciduria. ACSF3 mutations may cause symptoms suggestive of intermediary metabolic disorders, including vomiting and diarrhea in children or neurological manifestations later in life [2]. Although symptomatic patients with seizures, memory impairment, psychiatric symptoms, and cognitive decline have been reported [2, 3, 4, 5], the clinical relevance of CMAMMA remains debated [6]. Because many patients are diagnosed only in adulthood after years of misdiagnosis, long‐term disturbances in metabolic flexibility and secondary cellular dysfunction have been proposed [7, 8, 9]. The pathophysiology of CMAMMA remains poorly understood [9, 10]. Accumulation of MA and MMA, together with adaptive mitochondrial energy mechanisms, likely contribute to disease development but do not fully explain the broad clinical spectrum [2, 8, 9, 11]. The biological significance of *ACSF3* extends beyond rare disease. A recent evolutionary genomics study identified a human‐specific regulatory variant (rs34590044‐A) in an enhancer region of *ACSF3*, which upregulates its expression, enhances mitochondrial activity, reduces MMA accumulation, and is associated with increased stature and basal metabolic rate (BMR) in anatomically modern humans, particularly under meat‐enriched, threonine‐rich diets [12, 13, 14]. This finding places *ACSF3*‐dependent mitochondrial metabolism at the intersection of human metabolic evolution and disease, and underscores that even partial loss of *ACSF3* function, as in CMAMMA, may profoundly disrupt the metabolic homeostasis that this gene has helped shape over hundreds of thousands of years. A recent case report further expanded the CMAMMA clinical spectrum by describing neonatal hyperinsulinemic hypoglycemia, with reduced lipoylation of the pyruvate dehydrogenase (PDH) and α‐ketoglutarate dehydrogenase (αKGDH) suggesting that impaired mitochondrial lipoic acid biosynthesis may contribute to diverse clinical presentations [12]. We recently showed that impaired metabolic flexibility and increased reliance on mitochondrial fatty acid oxidation in ACSF3‐deficient fibroblasts are associated with dysregulated mitochondrial dynamics and enhanced mitochondrial fragmentation [7]. Here, we describe a young adult with fatigue, irritable bowel symptoms, and anxiety who underwent a long diagnostic journey before receiving a diagnosis of CMAMMA, and we investigate the effects of an *ACSF3* variant on mitochondrial metabolism in patient‐derived fibroblasts.

## 2 Results

### 2.1 Genetic Testing

Targeted genetic testing was performed as part of routine clinical care after appropriate patient consent. DNA was isolated from EDTA blood samples and sequencing of the following genes was performed: *MMUT*, *MMAA*, *MMAB*, *MCEE*, *MMADHC*, *ALDH6A1*, and *ACSF3*. The diagnosis was established using Alamut Visual 2.0 and comparison with ClinVar and HGMD databases.

### 2.2 Cell Culture, Protein Homogenates, Western Blot Analysis, and PDH Enzyme Testing

Experiments were performed with fibroblasts from three healthy controls (Coriell Institute: GM23964, GM23976, and GM08399) and the CMAMMA patient. Patient fibroblasts were obtained after signed informed consent. Cells were grown and homogenized in RIPA buffer with protease and phosphatase inhibitors. Total protein concentration was measured by BCA assay. Protein homogenates (25 and 50 μg) were separated by SDS‐PAGE and transferred to nitrocellulose. Antibodies used: anti‐malonylated lysine (Cell Signaling Technology, 1:1000), anti‐lipoic acid (Abcam, 1:2000), anti‐ACSF3 (Invitrogen, 1:1000), anti‐OXPHOS (Abcam, 1:250), and anti‐β‐actin as loading control. HRP‐conjugated secondary antibodies were used at 1:5000. Signals were detected and quantified with a Fusion Fx Analyzer and ImageJ. PDH enzyme activity was measured using the PDH enzyme assay Kit (MAK567‐1KT, Sigma‐Aldrich) per manufacturer's instructions using 30 mg protein.

### 2.3 Statistical Analysis and Use of AI Tools

In designing and reporting our experiments, we followed the current recommendations for distinguishing biological from technical replication in cell‐based studies [15, 16, 17, 18]. All experiments were performed using primary fibroblasts derived from a single CMAMMA patient and the corresponding control cell line. Biological replicates (*n* = 4) were generated by independently culturing the cells on three separate occasions. For each biological replicate, cells were cultured independently, lysed separately, and processed independently for the respective biochemical analyses. When technical replicates were performed, the corresponding measurements were averaged and used to obtain a single value for each biological replicate. Statistical analyses were therefore conducted using the independent biological replicates as the experimental unit, in accordance with current recommendations distinguishing biological from technical replication in cell‐based experiments.

Data are presented as means ± SEM. Significance was assessed by unpaired Student's *t*‐tests using GraphPad Prism 6.0 (GraphPad Software, San Diego California USA); *p* < 0.05 was considered significant. The AIHub tool of University of Freiburg was used for the grammatical correction of the text.

## 3 Results

### 3.1 Case Report

The patient is a 32‐year‐old German man, born at term to non‐consanguineous parents after an uneventful pregnancy. Early psychomotor development was largely normal, except for a speech delay during childhood that resolved with logopedic therapy. Mild short‐term memory difficulties during school age were also reported. No relevant family history of metabolic, neurological, or gastrointestinal disorders was known.

From adolescence (approximately age 15), the patient developed pronounced gastrointestinal symptoms including painful intestinal spasms, loud bowel sounds, diarrhea, and marked stress sensitivity, significantly impairing quality of life. Routine laboratory investigations by general practitioners found no organic cause, and irritable bowel syndrome was assumed. No metabolic investigations were performed. At age 20, the patient independently began supplementation with a multivitamin preparation and magnesium (250 mg/day), reporting improvement in fatigue, sleep quality, and abdominal symptoms.

At age 25, the patient independently underwent urinary testing for vitamin B12 deficiency. While serum B12 was normal, urinary MMA was markedly elevated (70.32 mg/g creatinine; reference < 2.4 mg/g creatinine), leading to referral to a specialized metabolic center. Repeated investigations consistently showed elevated MMA with normal B12 and homocysteine. In October 2021, serum MMA was 8007 nmol/L and urinary MMA 95 mg/g creatinine. MMA levels were subsequently monitored annually (Table 1).

**TABLE 1:** Measurements of methylmalonic acid in blood at different time points.

	MMA serum	MMA urin	MA urin	Homocystein	Coenzyme Q10
Dec 2017		> 70 mg/g crea (< 2.4)			
Jan 2018				9.6 μmol/l (< 12 μmol/l)	
June 2021		81.1 nmol/mol Hb (< 3.7)			
Oct 2021	8007 nmol/L (50–300 nmol/L)	95 mg/g crea (< 4mg/g)			
Sept 2022		105 mmol/mol crea (0–18)	6 mmol/mol crea (0–28)		
Sept 2023		104 mmol/mol crea (0–18)	5 mmol/mol crea (0–28)		
Jan 2024					0.42 mg/l (0.64–2.16 mg/l)
Sept 2024		86 mmol/mol crea (0–18)	9 mmol/mol crea (0–28)		

*Note:* Reference values are reported in brackets. Blood samples were analyzed from different laboratories. Values in brackets represent the reference values of the diagnostic laboratories where the analyses were performed. Amino acid amount above reference values is highlighted in red. Amino acid amount below reference values is highlighted in blue.

Abbreviations: Co‐Q10, coenzyme Q10; crea, creatinine; MA, malonic acid; MMA, methylmalonic acid.

Molecular genetic testing at age 29 identified a homozygous pathogenic variant in *ACSF3* (c.1672C>T; p.Arg558Trp), confirming CMAMMA. No additional pathogenic variants were detected. Despite repeated investigations, malonic acid was never detected in urine.

Following diagnosis, a moderately protein‐restricted, higher‐carbohydrate diet was recommended; however, this worsened gastrointestinal symptoms and anxiety. Increased protein intake led to rapid symptom improvement (Table 2). In an attempt to alleviate symptoms, the patient (height: 188 cm; body weight: 81.8 kg) systematically evaluated: (i) a low‐protein diet (60 g/day), (ii) a high‐protein diet (224 g/day), and (iii) a moderate‐protein diet (73 g/day) with glutamine supplementation (20 g/day). Dietary interventions were independently initiated by the patient and part of a controlled clinical protocol. Each dietary intervention was performed over 2 months. The greatest improvement in intestinal spasms, stress sensitivity, and anxiety occurred during the third intervention. Across all regimens, amino acid profiling showed consistently elevated plasma glutamine and histidine. No adverse events were reported by the patient.

**TABLE 2:** Amino acid analysis under different protein intake.

Amino acids	Reference values	Unit	60 g prot/day	223 g prot/day	73 g prot/day +20 g glutamine/day
Essential amino acids					
Leucine	43–180	nmol/mL	130.6	142.9	116.3
Isoleucine	24–98	nmol/mL	632	75.3	57.9
Threonine	62–170	nmol/mL	162	164.3	112.5
Valine	90–285	nmol/mL	241.7	282.9	210.7
Lysine	90–320	nmol/mL	188.1	186.3	149
Methionine	8–26	nmol/mL	25.3	23.4	19.6
Phenylalanine	30–73	nmol/mL	62.4	63	58.5
Triptophan	21–48	nmol/mL	74.1	49	49.8
Histidine	38–86	nmol/mL	89.7	93.5	74.4
Nonessential amino acids					
Glycine	135–375	nmol/mL	410.8	307.1	304.2
Alanine	159–464	nmol/mL	435.9	333.5	331.7
Serine	90–210	nmol/mL	220.7	170.2	159.3
Arginine	5–55	nmol/ml	33.3	44.8	18.3
Tyrosine	25–95	nmol/mL	64.2	59	58.2
Proline	76–300	nmol/mL	289.9	288.9	224.8
Glutamate	95–205	nmol/mL	150.5	147	180.3
Glutamine	180–440	nmol/mL	675.6	549.3	641.6
Aspartate	35–180	nmol/mL	146.1	137	122.4
Asparagine	47–97	nmol/mL	63.4	175.7	72.6
Non‐proteinogenic amino acids					
Citrulline	11–38	nmol/mL	35.1	48.4	43.6
Taurine	115–275	nmol/mL	131.6	266.8	111.8
Ornithine	50–160	nmol/mL	163.1	45.3	120.3

*Note:* Amino acid amount above reference values is highlighted in red. Amino acid amount below reference values is highlighted in blue.

The patient reports that gastrointestinal symptoms profoundly affected his quality of life from adolescence through his 20s (Figure S1B). Self‐initiated multivitamin and magnesium supplementation at age 20 provided partial relief. Following diagnosis, he found that protein restriction worsened symptoms, while higher protein intake combined with glutamine (20 g/day), riboflavin (200–400 mg/day), acetyl‐l‐carnitine, and magnesium substantially improved abdominal pain, anxiety, and overall well‐being. He highlights riboflavin as particularly effective for reducing stress sensitivity. The patient values the diagnosis for enabling targeted symptom management but expresses concern about the lack of disease‐specific treatment guidelines and long‐term prognosis.

### 3.2 Deficiency of ACSF3 Protein Severely Affects Lipoylation and Pyruvate Oxidation in Patient Fibroblasts

**FIGURE 1:**
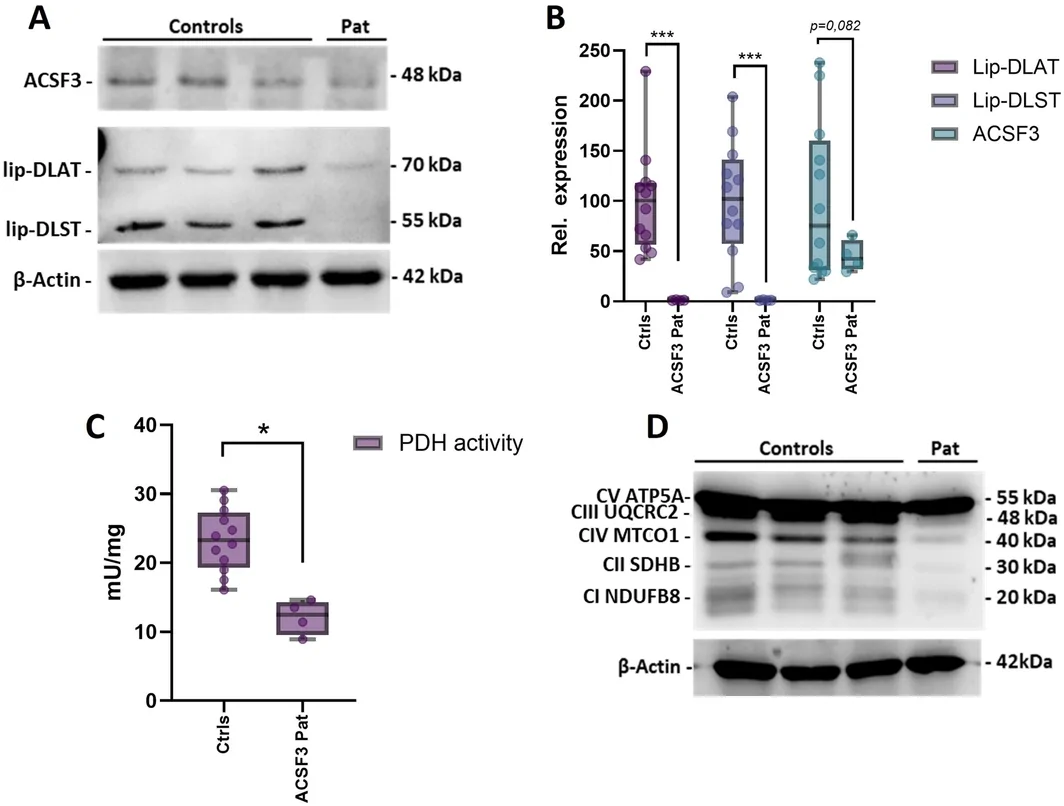
Effects of ASCF3 deficiency on mitochondrial proteins and pyruvate oxidation. (A) ACSF3 protein and lipoylation degree measured on the DLAT and DLST subunits of the PDC and αKGDH, respectively. (B) Quantification of the protein expression. (C) Pyruvate dehydrogenase enzyme activity. (D) OXPHOS; CI: Complex I—NDUFB8; CII: Complex II—SDHB; CIII: Complex III—MTCO1; CIV: Complex IV—UQCRC2; CV: Complex V—ATP5A. Western blots are representative of *n* = 3 biological replicates for healthy control and one patient's cell line. The values are mean ± SEM of four independent experiments (four replicates). Values denoted by * and *** were considered significant if *p* < 0.05 or *p* < 0.0005, respectively (Student's *t*‐test).

Global malonylation levels were analyzed in fibroblast lysates using a pan‐antibody recognizing malonylated lysine residues. Although a quantification of the malonylation degree was not performed, results seemed to show a selective increase in specific protein subsets (Figure S1C), suggesting redistribution rather than uniform upregulation. ACSF3 protein expression was only moderately reduced (45.1% ± 6.73%; *p* = 0.082) (Figure 1B).

OXPHOS complex analysis revealed a pronounced reduction of Complexes I, II, III, and IV, with complex V unaffected (Figure 1D). Lipoylation of PDC and αKGDH subunits was severely reduced, to 24.97% ± 3.25% (*p* = 0.000361) and 1.15% ± 0.12% (*p* = 0.000416), respectively (Figure 1A,B). Consistent with reduced lipoylation, PDH activity was significantly lower in fibroblasts of this patient (12.1 ± 2.05 mU/mg) compared to controls (23.3 ± 1.32 mU/mg; *p* = 0.0246) (Figure 1C). These cellular findings are particularly noteworthy in light of recent evolutionary evidence demonstrating that elevated *ACSF3* expression, driven by the human‐specific variant rs34590044‐A, enhances mitochondrial respiratory activity and reduces MMA accumulation in liver cells [14]. Our data in this patient fibroblasts represent, in essence, the pathological mirror image of that evolutionary gain‐of‐function: where the ancestral *ACSF3* variant supported metabolic homeostasis across human evolution, loss‐of‐function mutations collapse the same mitochondrial machinery, leading to respiratory chain deficiency and impaired energy substrate oxidation [9].

## 4 Discussion

CMAMMA is a rare disease caused by *ACSF3* variants and characterized by accumulation of MA and MMA [1]. Its clinical relevance remains controversial [6], and late symptom onset suggests chronic, progressive metabolic dysfunction [2, 7, 8, 9, 11]. A strength of this report is the detailed longitudinal characterization of clinical course, biochemical markers, and genetic findings, with systematic documentation of dietary interventions and patient‐reported outcomes. Limitations include the non‐controlled nature of dietary interventions and reliance on patient self‐report without standardized scales.

Placed in a broader biological context, the pathogenicity of *ACSF3* mutations gains further significance from the recent finding that *ACSF3* has been subject to positive selection in anatomically modern humans. Zhang et al. demonstrated that a human‐specific regulatory variant (rs34590044‐A), emerging approximately 653 000 years ago and under strong positive selection over the past 20 000 years, upregulates *ACSF3* expression, enhances mitochondrial respiratory capacity, and reduces MMA accumulation, effects that translated into greater body length and higher energy expenditure in mice fed threonine‐rich, meat‐based diets [14]. This evolutionary model implies that the *ACSF3*‐mtFAS axis has been critical for sustaining the elevated metabolic demands of anatomically modern humans consuming animal protein. Conversely, our patient's data illustrate what happens when this axis is disrupted by a coding variant. Indeed, MMA accumulates, mitochondrial respiratory complexes are depleted, and the organism's capacity to oxidize dietary substrates, particularly carbohydrates via PDH, is severely compromised. The symptomatic improvement observed with higher protein and essential amino acid intake, rather than carbohydrate loading, is fully consistent with the evolutionary logic identified by Zhang et al., in that *ACSF3* function is most critical precisely when the diet is rich in the essential amino acids, including threonine, that characterize meat‐based nutrition [13, 14].

Although serum malonic acid was never detected, altered lysine malonylation in patient fibroblasts supports metabolic dysregulation in ACSF3‐deficient cells. Previous studies showed that exogenous malonic acid exposure increased lysine malonylation in ACSF3 knockout cells [19]. Interestingly, Zhang et al. reported that CRISPR‐mediated suppression of *ACSF3* in hepatocytes (HepG2 cells) did not significantly alter malonate, malonyl‐CoA, or lysine malonylation, but did elevate MMA, suggesting cell‐type‐specific consequences of ACSF3 deficiency. Our patient's fibroblasts seemed to show a selective increase in specific protein subsets (Figure S1C), suggesting redistribution rather than uniform upregulation, indicating that the malonylation consequences of *ACSF3* loss may be tissue‐dependent and appear progressively over time.

The connection between mitochondrial dysfunction and neurodegeneration is well established [20]. Given the neurological manifestations in adult CMAMMA patients [2, 8], it is plausible that the enteric nervous system (ENS) may experience similar metabolic alterations. Previous studies have implicated mitochondrial DNA alterations in irritable bowel syndrome, suggesting mitochondrial diseases should be considered in patients with intestinal motility disorders [21, 22].

The dramatic reduction in lipoylation of PDC and αKGDH subunits, also independently observed by Gragnaniello et al. [12] in a neonatal CMAMMA patient presenting with hyperinsulinemic hypoglycemia, indicates that mitochondrial ACC1 cannot fully compensate for ACSF3 loss, suggesting these enzymes act synergistically and cannot substitute for each other's function [23]. The convergence of severely impaired lipoylation across independent CMAMMA cases of different ages and phenotypes reinforces that reduced mtFAS‐dependent lipoic acid synthesis is a core biochemical feature of ACSF3 deficiency. Reduced lipoylation was functionally reflected in a 50% decrease in PDH activity in our patient. In the neonatal case reported by Gragnaniello et al., the same impairment was proposed to contribute to dysregulated insulin secretion, since lipoic acid plays a known modulatory role in pancreatic β‐cell function.

Following diagnosis, the patient adopted a protein‐restricted, carbohydrate‐rich diet based on methylmalonic aciduria guidelines, as no CMAMMA‐specific guidelines exist [24, 25]. Symptoms worsened, accompanied by accumulation of potentially toxic glycine and serine [26]. We hypothesize that increased carbohydrate load, hindered by reduced PDH activity, enhances serine and glycine biosynthesis from glucose (Figure S1A) [27] while impairing the glycine cleavage system [28]. Reducing carbohydrates while increasing protein normalized amino acid levels and improved symptoms, likely by providing anaplerotic substrates for the citric acid cycle [29]. This dietary response pattern aligns with the evolutionary framework proposed by Zhang et al., in which *ACSF3* function is optimally engaged by protein‐rich, meat‐based diets. Glutamine remained elevated regardless of dietary intervention, likely due to redirection of excess α‐ketoglutarate toward endogenous glutamine synthesis secondary to partial αKGDH dysfunction [27].

## 5 Conclusions

We describe a symptomatic CMAMMA patient with fatigue and irritable bowel syndrome, expanding the clinical spectrum of the disease. Our case reinforces that CMAMMA is a clinically heterogeneous disorder whose manifestations span from the neonatal period to adulthood and affect multiple organ systems, with severely reduced lipoylation of PDC and αKGDH subunits as a unifying biochemical hallmark. Abdominal symptoms emerged during puberty but remained undiagnosed for years, illustrating that CMAMMA is easily overlooked when not included in standard diagnostic algorithms for fatigue and gastrointestinal presentations. Viewed through the lens of the recently described evolutionary model of *ACSF3* [13, 14], the pathophysiology of CMAMMA reflects a breakdown of a metabolic axis that has been under positive selection in modern humans for hundreds of thousands of years, one optimized for high‐protein, meat‐based nutrition and elevated mitochondrial energy homeostasis. The observation that our patient improved most markedly on a higher‐protein diet with essential amino acid support is consistent with this evolutionary framework and points to the value of prospective, CMAMMA‐specific dietary studies to inform evidence‐based nutritional recommendations for this distinct disorder. Nothing is currently known about riboflavin use in CMAMMA. Ongoing studies in patient‐derived cell lines aim to evaluate the effects of riboflavin and amino acid load on mitochondrial energy homeostasis, with the goal of informing therapeutic strategies for affected individuals.

## Author Contributions

**Sara Tucci:** conceptualization, resources, data curation, writing – original draft preparation, project administration, supervision. **Khaled Alatibi:** formal analysis, investigation. **Martin J. Hug:** writing – review and editing. All authors have read and agreed to the published version of the manuscript.

## Funding

This work was supported by the University of Freiburg.

## Conflicts of Interest

The authors declare no conflicts of interest.

## Supporting information


**Figure S1:** (A) Simplified schematic representation and working hypothesis of amino acid metabolism in CMAMMA. Cellular localization of the metabolic pathways and involvement of all additional pathways are not presented. The schematic representation was made using Biorender (https://www.biorender.com/). (B) Timeline with relevant information for the diagnosis of the patient. The schematic representation was made using Biorender (https://www.biorender.com/). (C) Global malonylation degree of cellular proteins detected with the anti‐malonylated lysine antibody. The arrows indicate protein bands that show a visibly stronger malonylation signal in patient‐derived fibroblasts compared to controls.

## Data Availability

The data that support the findings of this study are available on request from the corresponding author. The data are not publicly available due to privacy or ethical restrictions.
